# Unravelling the Role of Iroquois Homeobox 4 and its Interplay with Androgen Receptor in Prostate Cancer

**DOI:** 10.21203/rs.3.rs-3295914/v1

**Published:** 2023-10-24

**Authors:** Panchadsaram Janaththani, Gregor Tevz, Achala Fernando, Adil Malik, Anja Rockstroh, Thomas Kryza, Carina Walpole, Leire Moya, Melanie Lehman, Colleen Nelson, The Australian Prostate Cancer BioResource, The Practical Consortium, Srilakshmi Srinivasan, Judith Clements, Jyotsna Batra

**Affiliations:** 1 School of Biomedical Sciences, Faculty of Health, Queensland University of Technology, Brisbane, Queensland, Australia.; 2 Translational Research Institute, Woolloongabba, Brisbane, Queensland, Australia.; 3 Florey Institute of Neuroscience and Mental Health, University of Melbourne, Parkville, Melbourne, Victoria.; 4 Centre for Genomics and Personalised Health, Queensland University of Technology, Brisbane, Queensland, Australia.; 5 Mater Research UQ, Translational Research Institute, Woolloongabba, Brisbane, Queensland, Australia.; 6 Vancouver Prostate Centre, Department of Urologic Sciences, University of British Columbia, Canada.; 7 The Institute of Cancer Research, London, SM2 5NG, UK.

**Keywords:** Prostate cancer, androgen signalling, Iroquois-Homeobox 4, genome-wide association studies, insertion-deletion polymorphism

## Abstract

Genome-wide association studies have linked Iroquois-Homeobox 4 (IRX4) as a robust expression quantitative-trait locus associated with prostate cancer (PCa) risk. However, the intricate mechanism and regulatory factors governing IRX4 expression in PCa remain poorly understood. Here, we unveil enrichment of androgen-responsive gene signatures in metastatic prostate tumors exhibiting heightened IRX4 expression. Furthermore, we uncover a novel interaction between IRX4 and the androgen receptor (AR) co-factor, FOXA1, suggesting that IRX4 modulates PCa cell behavior through AR cistrome alteration. Remarkably, we identified a distinctive short insertion-deletion polymorphism (INDEL), upstream of the IRX4 gene that differentially regulates IRX4 expression through the disruption of AR binding. This INDEL emerges as the most significant PCa risk-associated variant within the *5p15* locus, in a genetic analysis involving 82,591 PCa cases and 61,213 controls and was associated with PCa survival in patients undergoing androgen-deprivation therapy. These studies suggest the potential of this INDEL as a prognostic biomarker for androgen therapy in PCa and IRX4 as a potential therapeutic target in combination with current clinical management.

## Introduction

Prostate cancer (PCa) is the second most frequently diagnosed cancer and the fifth leading cause of cancer death in men ^1^. It is well established that the Androgen Receptor (AR) plays a critical role in this hormone-stimulated disease. Androgen deprivation therapy (ADT), as well as androgen targeted therapy (ATT), are still the mainstream treatments for advanced disease ^2,3^. Although most patients initially respond, subsequently, they can develop resistance and progress to Castration Resistant PCa (CRPC) ^4^. This resistance is mediated by sustained AR signalling, genomic alterations at the *AR* locus and dysregulation of AR transcriptional cofactors ^5–7^. Therefore, AR-axis ^8^ or AR-coregulators such as ERG and FOXA1 ^9,10^ are being explored as potential therapeutic strategies to improve survival in CRPC patients. Genetic predisposition is a significant factor contributing to the risk of PCa ^11–13^. Through Genome-Wide Association Studies (GWAS), the rs12653946 SNP at the *5p15* locus was found to be significantly associated with PCa risk in multi-ethnic populations ^14–16^. Of note, the expression of a proximal protein coding gene Iroquois Homeobox 4 (*IRX4)* was correlated with the rs12653946 SNP genotype in prostate samples ^17^.

Iroquois-Homeobox 4 (IRX4) belongs to the IRX family, encoding a highly conserved homeodomain-containing transcription factor which plays fundamental pre-patterning roles in diverse developmental processes ^17^. The *Homo sapiens IRX* genes are organized as two clusters containing three genes each; *IRX1, 2* and *4* cluster on chromosome 5 and *IRX3, 5* and *6* on chromosome 16, albeit separated by large intergenic regions. Their deregulation has been implicated in various cancers: *IRX1 gene* hypomethylation is a potential molecular biomarker for lung metastasis; *IRX2* is an oncogene in osteosarcoma; *IRX3* is overexpressed in colorectal cancer; *IRX4* is a predictor of outcome in oral squamous cell carcinoma and *IRX5* is a cell cycle regulator in multiple cancers ^18–20^. *IRX4* had been reported to suppress PCa growth by interacting with the Vitamin D Receptor ^21^. A recent study has suggested that epigenetic inactivation of *IRX4* promotes pancreatic tumorigenesis ^22^. Conversely, IRX4 had been recently identified as a potential oncogenic transcription factor in non-small cell lung cancer ^23^. IRX4 also was detected at high levels in plasma samples of breast cancer patients ^24^. Even though evidence is accumulating to show the regulation of cell proliferation, apoptosis, angiogenesis and metastasis by different IRXs ^25,26^, the transcriptome affected by these transcription factors is still not fully understood.

This study aimed to elucidate the functional role of IRX4, investigate its transcriptome, and explore its co-factors to provide crucial insights into the clinical relevance of IRX4 in PCa. We discovered a distinctive INDEL located upstream of the *IRX4* gene, which disrupts AR binding and regulates IRX4 expression. This INDEL was the most significant PCa risk-associated within the *5p15* locus. Our findings suggest the potential of this INDEL as a prognostic biomarker for guiding androgen therapy in PCa.

## Results

### *IRX4* is overexpressed in PCa

**Figure 1:**
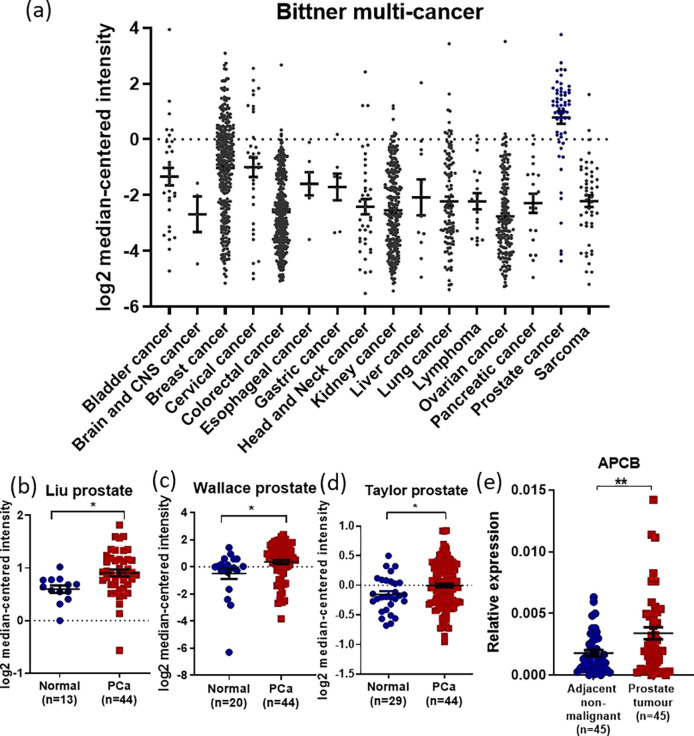
*IRX4* is overexpressed in prostate cancer tissues. **(a)** Higher expression of *IRX4* is detected in prostate cancer samples, followed by breast and cervical cancer, compared to other types of cancer, (Mean ± SD, Source – Oncomine, Bittner multicancer dataset, Bladder cancer = 32, Brain and CNS cancer = 4, Breast cancer = 328, Cervical cancer = 35, Colorectal cancer = 330, Esophageal cancer = 7, Gastric cancer = 7, Head and neck cancer = 41, Kidney cancer = 254, Liver cancer = 11, Lung cancer = 107, Lymphoma = 19, Ovarian cancer = 166, Pancreatic cancer = 19, Prostate cancer = 59 and Sarcoma = 49). *IRX4* is highly expressed in prostate tumour samples compared to the normal prostate in **(b)** Liu prostate (normal = 13, tumour = 44) **(c)** Wallace prostate (normal = 20, tumour =44). (d) Taylor prostate (normal = 29, tumour = 44) (Source – Oncomine, Mean±SEM, student’s t-test, *p<0.05) and **(d)** APCB (Australian Prostate Cancer BioResource) samples (n=45) (Mean±SEM, Wilcoxon signed-rank test, **p<0.01).

To assess the clinical significance of *IRX4* expression in PCa, we first analyzed publicly available cancer datasets for *IRX4* gene expression. Higher expression of *IRX4* was observed in PCa compared to other types of cancer (Oncomine ^27^; Bittner multicancer dataset) (Figure 1a). Moreover, higher *IRX4* expression was also observed in prostate tumour samples compared to normal prostate tissues in the Liu ^28^, Wallace ^29^ and Taylor ^30^ prostate datasets (Figure 1b, c and d). A similar trend was observed in Grasso ^31^, Arredouani ^32^ and Vanaja ^33^ prostate datasets, although the results were not statistically significant (Supplementary Figure 1). This was further validated using our own PCa FFPE tissues, in which *IRX4* gene expression was higher in tumour samples compared to adjacent non-malignant tissues (N=45, Australian Prostate Cancer BioResource, APCB, Figure 1e).

### *IRX4* knockdown suppresses proliferation, migration and invasion of PCa cells

**Figure 2:**
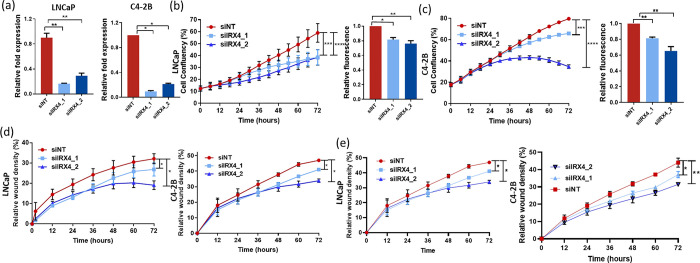
*IRX4* knockdown reduces prostate cancer cell proliferation and migration *in vitro*. **(a)** siRNA mediated knockdown of *IRX4* exhibited more than 75% knockdown efficiency at mRNA level on transfection in LNCaP and C42B cells (n=3, mean±SEM, Kruskal-Wallis test with Dunn’s multiple comparisons test, *p<0.05, **p<0.01). **(b)** *IRX4* knockdown in LNCaP cells reduced cell proliferation compared to non-targeting siRNA (siNT) transfection (n=3, mean±SEM, Friedman test with Dunn’s multiple comparisons test, ***p<0.001, ****p<0.0001) and exhibited lower fluorescent measurements compared to control cells at 72 hours of transfection, reflecting a reduction in proliferation in siIRX4 transfected cells (n=3, mean±SEM, Kruskal-Wallis test with Dunn’s multiple comparisons test, *p<0.05, **p<0.01). **(c)** *IRX4* knockdown reduced the proliferation of C4-2B cells compared to siNT control as measured by IncuCyte Live Cell Imaging system (n=3, mean±SEM, Friedman test with Dunn’s multiple comparisons test, **p<0.01, ***p<0.001) and cyQUANT assay (n=3, mean±SEM, Kruskal-Wallis test with Dunn’s multiple comparisons test, **p<0.01). **(d)** *IRX4* knockdown in LNCaP and C42B cells reduced migration towards wound closure compared to control (siNT) cell transfection (n=3, mean±SEM, Friedman test with Dunn’s multiple comparisons test, *p<0.05). **(e)** *IRX4* knockdown reduced cell invasion in both LNCaP and C42B cells, respectively (n=3, mean±SEM, Friedman test with Dunn’s multiple comparisons test, *p<0.05).

To understand the functional role of IRX4 in PCa, knockdown experiments were performed using siRNA targeting IRX4 in LNCaP and C4-2B PCa cells (Supplementary Figure 2a). Efficient knockdown (>80%) of *IRX4* expression was achieved in both cell lines (Figure 2a). Following IRX4 knockdown, a significant reduction in cell proliferation was observed in LNCaP cells compared to the non-targeting siRNA control (siNT), as assessed by the IncuCyte Live Cell Imaging System and cyQUANT assay at 72 hours post-transfection (Figure 2b). Similarly, IRX4 knockdown in C4-2B cells also resulted in decreased proliferation (Figure 2c).

Furthermore, the impact of IRX4 knockdown on cell migration and invasion was assessed. Scratch assays revealed a notable decrease in cell migration upon IRX4 knockdown in both LNCaP and C4-2B cells (Figure 2d). In addition, the Matrigel invasion assay demonstrated a significant reduction in cell invasion following IRX4 knockdown (Figure 2e). These findings highlight the functional role of IRX4 in regulating crucial cellular processes in PCa.

### *IRX4* regulates cellular pathways and modulates AR transcriptome

**Figure 3:**
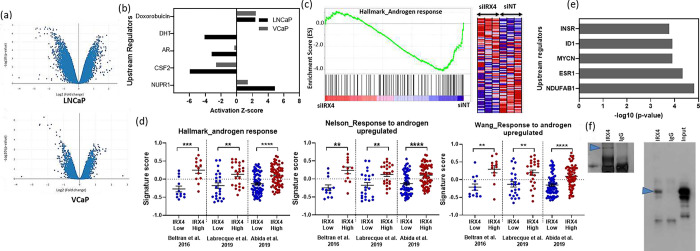
IRX4 mediates effective androgen signalling. **(a)** represents the volcano plot for the IRX4 regulated genes in LNCaP and VCaP cells respectively. The array contained 23250 genes and 1996 genes were found to be differentially regulated by *IRX4* knockdown in LNCaP cells, while 354 genes were identified to be differentially regulated in VCaP. The volcano plot was graphed using the log2 (fold change) and the −log10 (p-value) in the x and y-axis respectively. Each ● represents a gene and the genes with a p-value ≤ 0.05 and fold change ≤ −1.5 or ≥ 1.5 are considered as differentially expressed genes. **(b)** Upstream regulator analysis of IRX4 regulated genes in both LNCaP and VCaP knockdown samples. **(c)**. Gene set enrichment analysis (GSEA) of *IRX4* gene signature of LNCaP cells and androgen response (Hallmark gene set). **(d)** Gene signature scoring identified high expression of androgen response upregulated gene signatures in patients with high *IRX4* expression across multiple metastatic PCa clinical datasets. (Low *IRX4* expression [less than Q1] and high *IRX4* expression [higher than Q3]). Mean±SEM, Mann-Whitney test, ** p<0.01, *** p<0.001, **** p<0.0001. **(e)** Upstream regulator analysis with the proteins identified to be interacting with IRX4 in LNCaP. **(f)** Representative Western blot analysis with an IRX4 antibody to confirm IRX4 (blue arrow) pulldown and FOXA1 antibody detected FOXA1 (blue arrow) only in IRX4 pull down sample at 49 kDa.

To gain insights into the underlying mechanisms of the above identified functional implications, we performed microarray analysis followed by Ingenuity Pathway Analysis (IPA) on differentially expressed genes in IRX4-depleted LNCaP and VCaP cells (Supplementary Figure 2a, 2b). Approximately 1900 genes were differentially regulated on *IRX4* knock down in LNCaP cells (687 genes up-regulated, 1309 down-regulated, Figure 3a, Supplementary Table 1). On the other hand, only 352 genes were found to be differentially regulated in VCaP cells (165 up-regulated and 187 down-regulated, Figure 3a, Supplementary Table 2). 202 common genes were found to be deregulated by *IRX4* knockdown in both LNCaP and VCaP cell lines (Supplementary Figure 3a), of which 195 were regulated by *IRX4* knockdown in the same direction in these cells. The microarray results were validated by qRT-PCR for twelve of the common genes regulated by *IRX4* in both LNCaP and VCaP cells (Supplementary Figure 3b).

As expected from the observations from the cell-based assays above canonical pathways involved in cell cycle were found to be deregulated on *IRX4* knockdown in LNCaP cells (Table 1). This included signalling by Rho Family GTPases, Cell cycle and chromosomal replication and RhoA signalling. Moreover, cell cycle was predicted as the topmost deregulated molecular and cellular function in these cells followed by cellular assembly and organization and cellular growth and proliferation (Table 1). Top canonical pathways regulated by the *IRX4* gene signature in VCaP cells included regulation of the epithelial-mesenchymal transition pathway, Human embryonic stem cell pluripotency and Factors promoting cardiogenesis in vertebrates (Table 1). The most likely deregulated molecular and cellular function in *IRX4* knockdown VCaP cells was cell death and survival, followed by cellular movement and cellular growth and proliferation (Table 1). The data obtained from our microarray analysis reflects the known roles of homeobox genes in developmental process, including differentiation, early embryonic patterning, cell-type specification, and organogenesis ^34^.

*Colony stimulating factor* (*CSF2*) was predicted to be one of the most likely inhibited upstream regulator in *IRX4* knockdown LNCaP and VCaP cells (Figure 3b, Supplementary Table 3 and 4). On the other hand, *Nuclear Protein 1* (*NUPR1*) was found to be the most likely activated upstream regulator in these *IRX4* knockdown LNCaP cells (Figure 3b). Remarkably, our analysis revealed that knockdown of *IRX4* in LNCaP cells resulted in the inhibition of key upstream regulators of androgen signalling, including AR (z = −3.174, p = 6.18 × 10^−9^) and dihydrotestosterone (DHT) (z = −4.094, p = 3.12 × 10^−7^) (Figure 3b, Supplementary Figure 4a). This suggests that IRX4 may play a role in modulating effective androgen signalling. Although the activation score for *AR* did not reach the cutoff value, VCaP cells exhibited a similar trend of inhibition upon *IRX4* knockdown, with a statistically significant overlap of genes between the two datasets (z = −0.317, p = 7.00 × 10^−4^) (Supplementary Figure 4b). Furthermore, the negative enrichment of androgen response genes in LNCaP cells was confirmed by Gene Set Enrichment Analysis (GSEA) of Hallmark gene signatures (Enrichment score = −1.32, p < 0.001) (Figure 3c), indicating the potential regulatory role of IRX4 in the androgen signalling pathway.

Additional evidence for the role of IRX4 in androgen signalling was observed through the significant enrichment of upregulated androgen response gene signatures in patients with high *IRX4* expression, as determined by Gene Set Variant Analysis (GSVA), across multiple clinical datasets of metastatic PCa tissues (Figure 3d). In contrast, no significant enrichment of these signatures was observed in clinical samples from primary tissue (Supplementary Figure 5), highlighting the potential relevance of IRX4 in aggressive disease.

### AR transcriptome is regulated by IRX4 via interacting with FOXA1

The above pathway analysis results prompted further investigation into the implications of IRX4 in androgen signalling and its role in PCa progression. Two potential mechanisms can be proposed: First, IRX4 may exert regulatory effects on the AR cistrome by directly modulating AR expression. Alternatively, IRX4 may interact directly or indirectly with other proteins/transcription factors involved in AR signalling, potentially influencing AR activity. *IRX4* knockdown didn’t have any effect on *AR* transcript levels as observed in the microarray data (fold change= −1.24, p-value = 0.24), rejecting first hypothesis. Moreover, previous studies on investigating AR interacting proteins did not identify IRX4 as a binding partner of AR in PCa cells, including LNCaP cells ^35,36^, suggesting the AR transcriptome regulation by IRX4 is not a result of direct interaction between these two transcription factors. Therefore, we hypothesised that IRX4 may modulate the AR cistrome by either competing with, or guiding, AR to its chromatin binding sites or this observation might be a consequence of IRX4 interaction with other AR binding partners and therefore modulating AR activity.

To identify the interacting partners of IRX4 in LNCaP cells, an immunoprecipitation (IP) assay was performed with LNCaP nuclear enriched cell lysate. IRX4 was successfully pulled down and detected with 19% – 31.1% coverage. The proteins identified at least in two replicates were considered for further analysis. A total of 86 unique proteins were identified in the IRX4 IP sample (Cut-off intensity > E+07) compared to IgG control (Supplementary Table 5). AR was not detected in the IRX4 IP samples, further confirming lack of direct AR and IRX4 interaction. IPA upstream regulator analysis with the proteins found to be interacting with IRX4 identified Estrogen Receptor 1 (ESR1), as one of the top regulators (Figure 3e, Supplementary Table 6), further suggesting that IRX4 may be involved in hormone-regulated pathways. It also identified that other cancer related transcription regulators, including MYCN, INSR, ETV5 and MYC as upstream regulators of the IRX4-interacting partners (Figure 3e, Supplementary Table 6). Interestingly, the pioneer AR interacting transcription factor, FOXA1, also known as hepatocyte nuclear factor 3α (HNF-3α) was detected in the IRX4 IP sample (Supplementary Table 5). The interaction between IRX4 and FOXA1 was further confirmed by Western blot analysis in an independent IP experiment (Figure 3f). These data support our hypothesis that IRX4 might play a role in altering the AR transcriptome/cistrome by interacting with its well-established co-factor FOXA1 ^9^.

### Androgen regulation of *IRX4* expression is mediated by an Insertion-Deletion Polymorphism (INDEL)

**Figure 4:**
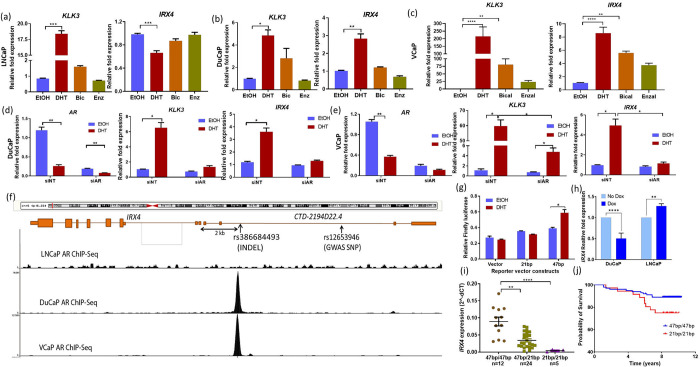
Expression of *IRX4* is regulated by androgens. (**a**), (**b**) and (**c)** qRT-PCR analysis showing downregulation of *IRX4* expression with 10 nM DHT treatment in LNCaP cells, while *IRX4* expression was upregulated by DHT treatment in DuCaP and VCaP cells. *KLK3* expression was used as a positive control to validate the androgen treatment. Anti-androgens, bicalutamide (10 μM) and enzalutamide (10 μM), inhibited the action of DHT (Mean±SD, n=3, Mann-Whitney test * P<0.05). (**d**) and (**e**) Transient knockdown of AR inhibited the androgen-mediated upregulation of *IRX4* in DuCaP and VCaP cells. The AR knockdown efficiency was measured by qRT-PCR analysis and the androgen treatment was validated by measuring *KLK3* expression (Mean±SD, n=3, Mann-Whitney test, *p<0.05). (**f**) Upper panel shows the genes at this locus including *IRX4*, which encompass the GWAS SNP, rs12653946 associated with prostate cancer risk. AR binding peaks were observed in VCaP and DuCaP cells, (~2kb upstream to *IRX4*), and not observed in the LNCaP cell line. (Cistrome finder (http://cistrome.org/finder derived/, Figure derived from UCSC Genome Browser). (**g**) LNCaP cells transfected with the 47bp allele had significantly higher luciferase activity with DHT treatment compared to the 21bp reporter construct. (n=2, mean±SEM, t-test, * p < 0.05). (**h**) Expression of *IRX4* lowered on disruption of 47 bp sequence in DuCaP cells and IRX4 expression was higher when 21 bp was replaced with the 47 bp sequence (n=2, mean±SEM, two-way ANOVA, ** p < 0.01, **** p < 0.0001). (**i**) The expression of *IRX4* was correlated with the INDEL and rs199577062 genotype. The risk SNP correlates with low expression of *IRX4*. The 21bp/21bp genotype correlates with lower transcript levels of *IRX4*. (APCB, mean±SD, Kruskal-Wallis test, ** p < 0.01, **** p < 0.0001). (**j**) The survival of the patients who underwent hormone deprivation therapy was lower in individuals harbouring the 21bp/21bp homozygous genotype, Hazards ratio (HR)=2.41, 95% CI=0.88–6.58, Log-rank test, p=0.04. (47bp/47bp = 101, 21bp/21bp = 36).

Following the characterization of IRX4’s functional and mechanistic role in PCa, we aimed to investigate the regulation of *IRX4* gene expression, particularly in response to anti-androgens such as bicalutamide and enzalutamide. To validate the effectiveness of androgen/anti-androgen treatments in the cell lines, we assessed relative *KLK3* expression (Figure 4a). In the LNCaP cell line, DHT treatment resulted in downregulation of *IRX4* expression (Figure 4a). However, in DuCaP and VCaP cells, we observed upregulation of *IRX4* expression following androgen treatment (Figure 4b and c), contrasting with the LNCaP cell line. The anti-androgens, bicalutamide and enzalutamide, effectively inhibited androgen action in both VCaP and DuCaP cells, providing further evidence for the androgen regulation of IRX4 (Figure 4b and c). Additionally, we conducted AR knockdown experiments in cell lines, where the androgen-mediated upregulation of IRX4 expression was suppressed, confirming the AR-mediated regulation of *IRX4* in DuCaP and VCaP cells (Figure 4d and e). Interestingly, AR binding peaks were observed upstream of *IRX4* gene in VCaP and DuCaP cell lines, while no AR binding was observed at this chromosomal locus in LNCaP AR ChIP-Seq data (Figure 4f) ^37,38^. Moreover, an ERG binding peak was observed at the same site in VCaP cells. This suggests that AR and ERG binding at this chromosome *5p15* locus may be important for expression regulation of *IRX4* by androgens in DuCaP and VCaP cells. Sequencing of the purified PCR products of this AR binding region revealed a rare type of Insertion-Deletion Polymorphism (INDEL, rs386684493), coinciding with the AR ChIP-peak in the upstream of *IRX4*. A stretch of 47bp sequence, which represent the consensus sequence was found to be replaced by a novel 21bp sequence in LNCaP cells. Effectively, the loss of this 47bp sequence would delete the binding site of AR and ERG and change how *IRX4* is regulated in androgen responsive cells.

### INDEL has a functional role in IRX4 gene regulation

The functional role of the identified INDEL in *IRX4* gene regulation was further investigated using reporter gene assay and CRISPR editing. To assess its impact, we employed differential promoter vectors containing either the 21bp or 47bp allele. Interestingly, the 47bp allele in the enhancer region exhibited higher luciferase activity when treated with DHT compared to the vehicle control, while the 21bp allele clones showed no difference in activity upon androgen treatment (Figure 4g).

To further explore the functional consequences of the INDEL, we utilized CRISPR/Cas9 genome editing to modify the 47bp allele in DuCaP cells, resulting in altered expression of *IRX4* (Figure 4h) and in response to androgen and anti-androgen treatments (Supplementary Figure 6a). Subsequently, we performed editing of the 21bp sequence, replacing it with the 47bp sequence in LNCaP cells, which led to increased expression of *IRX4* (Figure 4h). Moreover, proliferation analysis in these CRISPR-edited cell line models, displayed lower proliferation when IRX4 expression was lowered on disruption of the 47bp allele in DuCaP cells and the proliferation was higher when the 21bp was replaced with the 47bp sequence in LNCaP cells (Supplementary Figure 6b). These findings support the notion that the INDEL at the *5p15* locus modulates IRX4 expression and cellular function. Notably, individuals with the 21bp/21bp homozygous genotype exhibited lower transcript levels of *IRX4* (Figure 4i), suggesting a potential functional relationship between the INDEL genotype and IRX4 expression and align with the ChiP-seq data on AR binding site at this locus.

### INDEL association with PCa risk and survival for patients treated with ADT

Risk association analysis of PCa GWAS loci in 82,591 cases and 61,213 controls of European ancestry has identified rs199577062 (INDEL - _/G, chr5: 1889346), to be the most significant PCa risk associated variant at this locus (OR – 1.10 (1.09–1.11), p-value 1.62E-27, MAF = 0.40, OncoArray Project - PRACTICAL Consortium ^39^) (Supplementary Figure 7). This SNP coincides with the last nucleotide of INDEL (chr5: 1889300–1889346) and since the probe is designed to the negative strand for this SNP, this will identify the INDEL genotype. This was further confirmed by genotyping the INDEL in 800 samples by PCR and then validating with the genotyping data for SNP rs19957702 from the OncoArray project ^11,39^. Interestingly, the 21bp/21bp genotype was associated with poor survival outcome in patients who underwent ADT, HR=2.41, 95% CI=0.88–6.58, Log-rank test, p=0.04 (Figure 4j).

**Figure 5:**
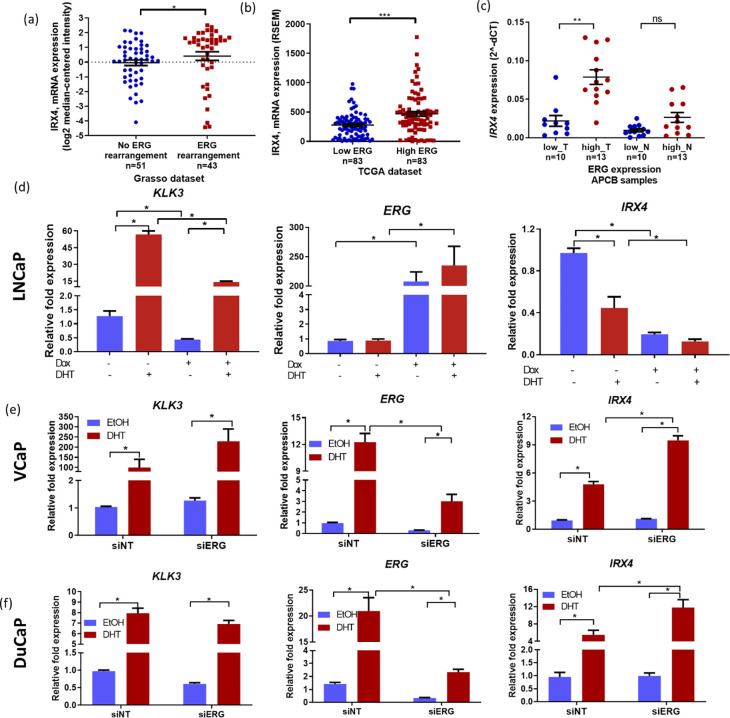
IRX4 expression correlates with ERG expression. *IRX4* is overexpressed in prostate tumour tissues with **(a)** ERG-rearrangement (Grasso dataset; Mean±SEM, Mann-Whitney test, * p < 0.05) and (b) with high ERG expression in TCGA samples (Low ERG expression [less than Q1] and high ERG expression [higher than Q3], Mean±SEM, Mann-Whitney test, *** p<0.001) **(c)** and APCB samples (T –Tumour, N – Adjacent non-malignant, Low = 10 [low ERG expression, less than Q1] and High = 12 [High ERG expression, higher than Q3], Mean±SEM, Mann-Whitney test, ** p<0.01, ns – nonsignificant.) **(d)** LNCaP-pIND21-ERG cells overexpressing ERG exhibited reduced *IRX4* expression, which was further reduced with DHT treatment. The DHT treatment was validated by checking *KLK3* expression and the ERG overexpression was validated by measuring the relative ERG mRNA expression levels (Mean±SD, n=3 biological replicates, Mann-Whitney test, * p<0.05). **(e)** and **(f)** *IRX4* expression was upregulated with 10 nM DHT in VCaP and DuCaP cells, and androgen mediated up-regulation of *IRX4* expression was further increased with ERG knockdown. *KLK3* expression was used as a positive control to validate the androgen treatment (n=3 biological replicates, Mean±SEM, Mann-Whitney test, *p<0.05).

Since the risk association of the chromosome *5p15* locus has been reported to be in the opposite direction with ERG fusion status in the previous studies ^40^, we explored the *IRX4* expression correlation with ERG expression. The expression of *IRX4* was found to be higher in ERG fusion positive PCa compared to ERG fusion negative samples (Grasso PCa data set, Figure 5a). Similarly, in the TCGA dataset, high *IRX4* expression correlates with high *ERG* expression (Figure 5b). In addition, ERG contribution to the up-regulation of *IRX4* expression is evident in the literature where *IRX4* is over-expressed in the primary and metastatic samples with the ERG fusion ^41^. We further confirmed the correlation between *IRX4* and ERG expression by qRT-PCR analysis in our PCa cohort tissues (APCB, Figure 5c). Similar to our observations in public datasets, high *IRX4* expression correlates with high ERG expression in prostate tumour samples (Figure 5c). Thus, we hypothesised that the difference in androgen responsiveness of *IRX4* expression observed in LNCaP cells in comparison to VCaP and DuCaP cells may be the result of low expression of ERG in the *TMPRSS2-ERG* fusion-negative LNCaP cell line. Interestingly, the expression of *IRX4* was down-regulated with ERG over expression, independent of androgen treatment (Figure 5d). To further understand the ERG-mediated regulation of *IRX4* in PCa cells, we determined the expression of *IRX4* in transient ERG knockdown VCaP and DuCaP cells (Figure 5e and f). *KLK3,* a key androgen regulated gene, expression was used as the positive control for DHT treatment (Figure 5d–f). Interestingly, the androgen mediated up-regulation of *KLK3* expression is further increased with ERG knockdown in VCaP cells, while no difference was observed with ERG knockdown on *KLK3* expression in DuCaP cells (Figure 5e and f). *IRX4* expression was upregulated with DHT treatment and further increased with the ERG knockdown in both VCaP and DuCaP cells, suggesting ERG may have a negative effect on androgen response of *IRX4* expression (Figure 5e and f).

## Discussion

The binding of steroidal androgens, testosterone and DHT to AR, causes AR receptor dimerization which enables the recruitment by androgen response elements on DNA and also the recruitment of a series of cofactors to regulate AR target genes. ADT/ATT is used for the treatment of PCa. Although there is initial efficacy of this treatment, most patients with advanced PCa eventually develop resistance and progress to CRPC, for which there is no curative therapy. Therefore, more targeted therapeutics are essential for the treatment of PCa. Our study is the first to identify IRX4 as a co-regulator of the AR transcriptome in PCa, likely through interacting with the FOXA1 transcription factor. AR, a member of the nuclear hormone receptor family of transcription factors, plays a vital role in the development and progression of the PCa. Hence, identification of IRX4 as a co-regulator of the AR transcriptome in this disease could provide new impetus for a more targeted PCa therapy.

Herein, we show a reduction in PCa cell proliferation with *IRX4* knockdown, which conflicts with the previously published data, in which *IRX4* knockdown was proposed to increase PCa cell proliferation ^21^. This could be due to the siRNAs used in these two studies to target different exons of the *IRX4* gene, and thus might target different splice variants/isoforms of the *IRX4* ^42^. Alternative splicing is reported as a common feature for genes encoding transcription factors with the expression of the spliced exons demonstrating tissue-specific expression ^43^. For example, another homeodomain gene HNF1B, which encodes for three variants A, B and C has been shown to have opposite functions, as variants A and B act as a transcriptional activator while variant C functions as a transcription repressor ^44^. Thus, using additional siRNAs targeting the variant containing the new exon will further confirm the results obtained in our studies. Similar to our observation in PCa cell proliferation, IRX4-positive ventricular progenitor cells were observed to have a high proliferation rate ^45^. Moreover, knockdown of another IRX family member, IRX5 also led to reduced proliferation in PCa cells ^46^. Additional studies using the overexpression models of different *IRX4* variants will identify their individual functions. Gene microarray analysis with *IRX4* knockdown in LNCaP and VCaP cells found cell cycle and EMT pathways to be modulated providing a plausible explanation for the differences in proliferation and invasive ability of PCa cells observed in our functional assays.

Interestingly, human embryonic stem cell pluripotency is one of the top deregulated pathways in *IRX4* knockdown VCaP microarray analysis. Published literature on *IRX4* as the marker of ventricular myocardium differentiation with multipotential ability to differentiate into ventricular myocytes, smooth muscle cells and endothelial cells ^45^, suggests similar genes and pathways may be regulated by *IRX4* in PCa cells. Even though, the number of genes regulated by *IRX4* knockdown is higher in LNCaP cells compared to VCaP cells, similar upstream regulators were predicted to be deregulated in both the LNCaP and VCaP cells including inhibition of *CSF2* and activation of *NUPR1*. Although not directly implicated in PCa, CSF2 overexpression has been reported to be associated with STAT5 phosphorylation, which is proposed as a potential therapeutic target for epithelial tumours such as prostate and breast cancer ^47^. The cellular stress responsive gene *NUPR1* has been implicated in context-dependent biological functions, which accounts for the oncogenic and a suppressive role of this protein in tumour growth.

Interestingly, the expression of AR regulated genes was influenced by *IRX4* knockdown in both cell types, with the effect being larger in LNCaP cells. The significant enrichment of androgen response gene signatures in patients with high *IRX4* expression across multiple metastatic PCa datasets suggest potential involvement of IRX4 in aggressive PCa. We further identified some common binding partners of AR and IRX4, including the pioneer co-factor, FOXA1, suggesting that the modulation of androgen signalling by IRX4 may be mediated via FOXA1. FOXA1 is known to remodel chromatin to allow genomic access by hormone transcription factors, including AR and the ER ^48^. This AR co-factor has been reported to promote tumour progression by inducing cell cycle pathways in androgen dependent PCa. A well-known homeobox protein, HOXB13, also has been reported to colocalize with FOXA1 and thereby reprogram AR binding sites in prostate tumour tissue ^49^.

In addition to identifying the intricacy of IRX4 and AR interaction we have established the functional role of the PCa risk associated INDEL, rs386684493, in androgen-mediated regulation of *IRX4* expression in an allele specific manner using CRISPR-edited cell line models, reporter gene assays and gene expression studies. This INDEL was previously reported to be the most strongly associated variant with PCa risk at the *5p15* locus in a Japanese population ^14^. Even though, Takata et al., performed a reporter gene assay with the INDEL alleles, they didn’t observe any differences, likely since androgen treatment was not included in the assay. As LNCaP cells have a missense mutation (T877A) in the ligand binding domain of the *AR* gene, which alters the binding characteristics of the AR ^50^, we performed a promoter vector assay in LNCaP cells. This analysis confirmed that the AR-mediated regulation of *IRX4* expression is independent of the AR gene mutation status in LNCaP. In addition, this study explored the function of the INDEL using CRISPR-edited cell line models for the first time, that verified the expression levels of *IRX4* transcript is dependent on the INDEL genotype. Proliferation analysis in these genome-edited cell lines were consistent with lower proliferation of PCa cells on IRX4 knockdown, indicating the role of IRX4 and INDEL in regulating crucial cellular processes in PCa. Whitington et al. also reported that this variant disrupts position weight matrices (PWMs) for AR, AR-FOXA1, NR3C1 and ETV1 ^51^. Some other SNPs which fall in the same linkage disequilibrium (LD) block with the INDEL, were also shown to have differential binding with nuclear proteins by Electrophoretic Mobility Shift Assay (EMSA) in a previous study ^21^, suggesting additional SNPs could also have an important functional role.

In addition to AR, we also observed FOXA1 and POL2RA binding at the INDEL in VCaP cells, while no such binding was observed in LNCaP cells (Supplementary Figure 8). Remarkably, binding of ETV1 and ERG, members of the ETS family of transcription factors, to the INDEL was observed in LNCaP and VCaP cells, respectively (Supplementary Figure 4). In order to identify other transcription factors binding to the alleles of the INDEL, the *in-silico* prediction tool, TFBIND, was utilised (cut-off score >=8) ^52^. There were only a few other transcription factors predicted to bind only to the 47bp allele of INDEL, including FOXA2 and SOX5 (Supplementary Table 7). On the other hand, only NKX2.5 was predicted to bind specifically to the 21bp allele of the INDEL. Interestingly, cardiac expression of IRX4 is reported to be modulated by NKX2.5 and the loss of this transcription factor in mice had resulted in lower levels of *IRX4* transcripts ^53^. On the other hand, the 47bp allele was predicted to bind to more proteins, including the developmental transcription factors, FOXA2 and SOX5. Interestingly, the 21bp/21bp genotype was associated with poor survival outcome for patients who underwent ADT in the APCB cohort. Additional survival analysis in a larger population will provide insights into the potential of this INDEL as a prediction tool for the outcome of hormone deprivation therapy. Moreover, detailed exploration of the interplay of IRX4, FOXA1 and AR at the cistromic level will identify the shared targets between these transcription factors and confirm the role of IRX4 in effective androgen signalling and as a potential therapeutic target for PCa management.

## Methods (To be included in the Supplementary)

### Analysis of IRX transcription factor expression in published data sets

Expression data for the IRX transcription factors in various cancer and normal tissues were downloaded from Oncomine ^27^ (excluded datasets with <20 samples), FireBrowse (Broad Institute) and cBioportal ^54^.

### Cell culture

Androgen responsive PCa cell lines (LNCaP, VCaP, DuCaP and C4-2B) were used in this study. All cell lines were obtained from the American Type Culture Collection (ATCC). All cell lines were grown in RPMI1640 media with no phenol red (Life Technologies) supplemented with 5% or 10% fetal bovine serum (FBS, Sigma). Cell lines were authenticated by Short Tandem Repeat (STR) profiling and tested negative for mycoplasma.

### siRNA mediated knockdown

Cells were transfected with siRNAs using the Lipofectamine^®^ RNAiMAX Transfection Reagent (Invitrogen, Catalog number – 13778150) according to the manufacturer’s instructions and incubated at 37 °C (5% CO2) for 72 hours and transfection efficiency was confirmed by qRT-PCR analysis. siRNAs used in this study were siIRX4 (Ambion, Catalog number – AM16708, s27097 and HSS121376), siERG (Ambion, Catalog No 4392420, s4811), siAR (Dharmacon, Catalog number - DHA- J-003400-07-0010) and non-targeting siRNA (Ambion, Silencer select no 1 siRNA, Catalog number – 4390843).

### Proliferation, Migration and Invasion assays

Proliferation assays were performed using the IncuCyte live cell imaging system (Essen Biosciences) with LNCaP cells (5000 cells/well) and C4-2B cells (2500 cells/well) in a 96 well plate transiently transfected with siRNAs using Lipofectamine^®^ RNAiMAX transfection reagent (Invitrogen, Catalog number – 13778150). Migration assays were performed in a poly-L-Ornithine (Sigma-Aldrich, Catalog number - P4957) coated 96-well ImageLock plate (Essen BioScience, Catalog number - 4379) and the cells were treated with mitomycin C at a concentration of 10 μg/mL (Sigma-Aldrich, Catalog number – M4287) for 2 hours. Uniform wounds were created in the wells using WoundMakerTM and each well was washed with culture media two times. Finally, 100 μL of media was added to the cells and the plate was placed in the IncuCyte (Essen BioScience). Two images per well were taken every two hours for three consecutive days and percentage of confluency and/or wound closure was measured by the IncuCyte integrated image analysis software. CyQuant NF assays (ThermoFisher Scientific, Catalog number – C35006) were performed in black plastic plates (Perkin Elmer Life Sciences, Catalog number - 6005182) according to the manufacturer’s instructions and fluorescence at 520 nm was measured after excitation at 480 nm using a microplate reader (FLUO Star Omega, BMG LAB TECH). The assays were performed with minimum of three technical replicates in at least three separate experiments. The data was plotted for relative confluency and/or relative wound closure (mean ± SEM) vs. time using Graph Pad Prism 7.0.

### Immunofluorescence (IF) analysis

IF analysis was performed to confirm the knockdown efficiency of siIRX4_1. Transient knockdown cells fixed with 4% PFA for 15 minutes at room temperature were permeabilized using PBS + 0.05 Triton X100 for 5min at room temperature. After three washes with PBS, cells were stained for F-Actin using Phalloidin-488 (1/40 in PBS). After washes to eliminate excess phalloidin, cells were saturated with PBS containing 3% BSA (30 minutes at room temperature), then incubated overnight at 4 °C with anti-IRX4 antibody (1/300 in PBS-3%BSA) and washed three times with PBS at room temperature, followed by incubation with anti-Rabbit IgG Secondary Antibody coupled with Alexa Fluor^®^ 563 conjugate (1/1000 in PBS-3%, 1h at RT). After three washes with PBS, cell nuclei were stained with DAPI and images were taken with an Olympus Inverted Fluorescence microscope.

### Microarray gene expression profiling and data analysis

For gene expression profiling, triplicates of each sample (LNCaP and VCaP cells with *IRX4* knockdown and non-targeting control) were analyzed on a custom 180k Agilent oligo microarray (ID032034, GPL25684) at the Australian Prostate Cancer Research Centre – Queensland (APCRC-Q). This array contains probes mapping to human protein-coding and non-coding loci; with probes designed for exons, 3’UTRs, 5’UTRs, intronic and intergenic regions. RNA was isolated using the RNA Mini Kit (Bioline) according to the manufacturer’s protocol, including an on-column DNAse treatment step. The integrity of the RNA samples for microarray analysis was confirmed using the Bioanalyser (RNA Integrity Number, RIN>9.5), while the concentration and purity of the RNA (A260/A280 > 2) samples were confirmed by Nanodrop1000 measurements. 150 ng RNA of each sample was amplified and labelled using the Agilent ‘Low Input Quick Amp Labeling Kit’ for One-Color Microarray-Based Gene Expression Analysis. Briefly, the RNA was reverse transcribed into cDNA using an oligo-dT/T7-promoter hybrid primer which introduced a T7 promoter region into the newly synthesised cDNA. Then *in vitro* transcription was performed using a T7 RNA polymerase, which simultaneously amplified the target material and incorporated cyanine 3-labeled CTP. cDNA synthesis and *in vitro* transcription was performed at 40 °C for 2 h, respectively. The labelled cDNA was purified using the RNeasy Mini Kit (Qiagen) and quantified on a NanoDrop1000. Finally, 1650 ng cRNA of each sample were hybridised at 65 °C for 17 h and the arrays subsequently scanned on an Agilent Microarray Scanner G2565CA. The microarray raw data were processed using the Agilent Feature Extraction Software (v10.7). A quantile between array normalization was applied and differential expression was determined using the Baysian adjusted t-statistic linear model of the ‘Linear Models for Microarray Data’ (LIMMA) package in R. p-values were corrected for a false discovery rate of 5% and gene expression levels are presented as log2 transformed intensity values. Normalized gene expression data from the experiment are annotated based on hg38/Ensembl.v.77/Gencode.v.21 and are ‘Minimum Information About a Microarray Experiment’ (MIAME) compliant. Genes that were significantly different between two groups were identified with an adjusted p-value of ≤0.05, and an average absolute fold change of >=1.5. For functional annotation and gene network analysis, filtered gene lists were examined using QIAGEN’s Ingenuity^®^ Pathway Analysis (IPA^®^, QIAGEN) and Gene Set Enrichment Analysis (GSEA, Broad Institute).

### Derivation of androgen response gene signature

Androgen regulated gene enrichment score was calculated using Gene Set Variation Analysis (GSVA) - (R-package) for gene signatures obtained from the GSEA (Nelson ^55^ and Wang ^55^ signatures).

### Immunoprecipitation (IP)

The nuclear enriched lysate from LNCaP cells was isolated using NE-PER nuclear and cytoplasmic extraction kit (ThermoFisher Scientific, Catalog number – 78833) according to the manufacturer’s instructions. Lysate was pre-cleared with 25 μL of SureBeads^™^ Protein G magnetic beads (BIO-RAD, Catalog number – 1614023) for 1 hour at 4 °C and the beads were removed three times using a magnetic stand. The extract was then incubated with 5 μL of 1mg/mL antibody for IgG or IRX4 (Abcam, Catalog number – ab123542) and 50 μL of protein G beads at 4 °C for overnight. Beads were washed with ice-cold PBS three times and the proteins were eluted with 100 μL nuclear extraction buffer by boiling the samples at 95 °C for 5 minutes.

### Mass spectrometry analysis

The mass spectrometry analysis was performed at the TRI Proteomics facility. The IP samples (5 μg) were boiled in sodium deoxycholate buffer (1% sodium deoxycholate, 10mM TCEP, 40mM 2CAA, 100mM Tris pH8.5) and diluted in H_2_O. Then the samples were digested with trypsin (0.1 μg) and incubated at 37 °C overnight and acidified with final formic acid concentration of 1%. The sodium deoxycholate precipitate was removed by centrifugation at 13000g for 10 minutes. The samples were then cleaned with C18 tips and resuspended in 0.1% formic acid, 3% acetonitrile to obtain 0.5 μg/μL concentration and finally the samples were sonicated for 5 minutes.

1 μg of samples were injected for the LC-MS/MS analysis onto a trap column (Easy LC THC164705 column (C18, 20 MM × 75um ID, 3um particle)) and then separated on an analytical column (Easy LC THCES803 column (C18, 500 MM × 50um ID, 2um particle)), The following LC gradient was applied: 3% B for 5 mins, 25B for 80 mins, 40B for 20 mins, 95%B for 1min, wash at 95% B for 10min, and back to 3% B in 1min. Data Analysis was performed with the following parameters on the Spectrum Mill B.05: Extract: Precursor MH+ 600–6000m/z, Retention time and m/z tolerance of +45sec, +1.4m/z, spectral similarity & RT & m/z merging, Xcalibur centroiding algorithm for profile mode data, max precursor of 6, min MS1 S/N of 25, find 12C precursor m/z. The search was done against Swissprot Human Nov 2014, trypsin digest, fix carbamidomethylation C, variable oxidized M, min matched peak intensity of 50%, Instrument ESI Reactive HCD, monoisotopic masses, precursor mass tolerance of + 20ppm, product mass tolerance of + 20ppm, max ambiguous precursor charge of 3, reversed database scores were calculated, discriminant scoring off, variable modification search mode, precursor mass shift range of −18 to 177kDa. The proteins identified in at least two replicates were considered for further analysis.

### *IRX4* expression analysis in patient tissues

Formalin fixed and paraffin-embedded (FFPE) blocks from prostate tumours and their adjacent non-cancer prostate were obtained for 50 patients from the Australian Prostate Cancer Bio-Resource (APCB) tumour bank. Tissue blocks were serially sectioned (20 μm sections) and total RNA was extracted. Briefly, macrodissection was performed using a sterile injection needle and deparaffinised using deparaffinisation solution (Qiagen) for the tumour areas marked by two pathologists. RNA was extracted using the miRNAs’ FFPE kit (Qiagen) according to the manufacturer’s instructions. RNA quality and quantity was measured using NanoDrop^™^1000 (Therma Scientific). First strand cDNA was synthesised using the SuperScript III reverse transcriptase kit (Invitrogen) for 0.5 – 1 μg of RNA. Relative mRNA levels determined by qRT-PCR were measured on ViiA^™^7 Real-Time PCR System (Applied Biosystems) using the SYBR Green (Invitrogen) method with gene-specific primers. *HPRT1* and *RPL32* were employed as internal controls. Relative expression compared to control was determined by the comparative CT (ΔΔCT) method.

### Androgen deprivation assay

LNCaP, VCaP and DuCaP cells seeded in RPMI1640 media (Life Technologies, Catalog number - 11835-030) supplemented with 5% fetal calf serum (FCS) and incubated at 37°C for 3 days. The medium was then replaced with androgen-depleted culture medium (RPMI1640) containing 5% charcoal-stripped serum (CSS). After 48 hours, the cells in CSS were supplemented with either 10 nM DHT or 10 nM DHT+10mM Bicalutamide/Enzalutamide or ethanol (EtOH, vehicle control) and incubated at 37°C for 48 hours.

### Genotyping of cell lines

PCR was carried out to amplify the AR/ERG binding region with 10 ng genomic DNA of cell lines using Platinum^®^Taq DNA Polymerase (Invitrogen, Catalog number - 11304011). The cycling parameters used were 95°C for 5 min, 35 cycles of 95°C for 15 sec, 60°C for 30 sec and 72°C for 1 min and final extension at 72°C for 10 min. The products were genotyped for genetic variation by running them on a 2% agarose gel as described in section 2.6. Sequencing of the PCR products was performed at the Australian Genome Research Facility (AGRF).

### Reporter Gene assay

The reporter vector constructs were established using a pGL3 promoter (pGL3-p) vector (Promega, Catalog number - E1761). Briefly, the primers were designed with overhangs containing the restriction sites for SacI and XhoI (restriction sites to be used in pGL3 promoter vector) to amplify the INDEL. Genomic DNA of DuCaP cells was used as a template for the 47bp sequence and LNCaP DNA was used as a template for the 21bp sequence. Both the pGL3–p vector and 21/47bp insert fragments were digested with SacI and XhoI, gel purified using Wizard^®^ Gel and PCR Clean-Up System (Promega) and ligated together using T4 DNA Ligase as per manufacturer’s instructions. Vector expression of inserts was then confirmed by restriction digest and sequencing prior to transfection into target cell lines. The LNCaP cells plated in six-well plates for 24 hours in CSS containing media were transfected with luciferase reporter plasmid and pRL-TK (Renilla Luciferase) using the Fugene 6 reagent (Roche) according to the manufacturer’s instructions. After 24 hours, cells were treated with either 10nM DHT or EtOH (Vehicle control). The cells were solubilized with lysis buffer after 24 hours and were treated with luciferase assay reagent (LAR II) to measure the firefly luciferase activity followed by addition of Stop and Glo reagent to measure the renilla luciferase activity in a luminometer according to the manufacturer’s instructions (Dual-Luciferase^®^ Reporter Assay and Dual-Luciferase^®^ Reporter 1000 Assay Systems, Promega). Renilla luciferase was used to normalise the recorded luciferase activity of the transfected cells.

### CRIPSR-Cas9-based gene editing

The MIT CRISPR tool (http://crispr.mit.edu/) was used to design guide RNA to 47bp and 21bp alleles (Supplementary Table 8). Guide RNA specific to 47bp or 21bp alleles were cloned into the doxycycline-inducible guide RNA vector Fgh1UTG (kindly provided by Dr Marco Herold Lab). LNCaP and DUCaP cells were infected with lentiviral Cas9 (pCas9Cherry, kindly provided by Dr Marco Herold and guide RNA cloned Fgh1UTG vector. To produce lentiviral particles, HEK293T cells were transiently transfected with vector DNA, along with the packaging plasmids pMDL, pRSV-rev and pVSVG, and the target plasmid (pCas9, Fgh1UTG) using Fugene6 HD transfection reagent (Promega) according to the manufacturer’s guidelines. Virus-containing supernatants were collected after 48 h and LNCaP and DuCaP cells were infected with the viral suspension in the presence of polybrene (8 μg/mL). After 24 h, viral transduced cells were sorted for cells positive for mCherry (pCas9) and EGFP (Fgh1UTG) to produce double-positive cells. The disruption or substitution of the allele sequences were then confirmed by Sanger Sequencing (AGRF).

### Statistical analysis

Assays were performed in three biological replicates. Data from qPCR experiments, functional assays and clinical data were statistically analyzed using GraphPad Prism 7.0. The comparison between two unpaired groups was analyzed by either student’s t-test or Mann-Whitney t-test and paired groups by Wilcoxon matched-pairs ranked test. More than two groups were analyzed using One-Way ANOVA OR Kruskal-Wallis test with Dunn’s multiple comparison test for unpaired samples and using the Friedman test for matched groups. The results were considered statistically significant if p <0.05. Kaplan–Meier analysis was performed to determine survival outcome using Kaplan Meier survival curves in GraphPad Prism for the APCB cohort.

## Supplementary Material

Supplement 1

## Figures and Tables

**Table 1:** Top canonical pathways and molecular and cellular functions deregulated in IRX4 knockdown LNCaP cells and VCaP cells

**LNCaP cells**	**Top Canonical Pathways**	**p-value**
	Signalling by Rho Family GTPases	5.10E-07
	Cell cycle control of chromosomal replication	7.53E-07
	RhoA signalling	1.07E-06
	**Molecular and Cellular Functions**	**p-value**
	Cell cycle	1.78E-03 – 4.60E-17
	Cellular assembly and organisation	1.89E-03 – 3.81E-16
	Cellular growth and proliferation	1.89E-03 – 1.98E-14
**VCaP cells**	**Top canonical pathways**	**p-value**
	Regulation of the Epithelial-Mesenchymal Transition Pathway	1.77E-04
	Human embryonic stem cell pluripotency	2.90E-04
	Factors promoting cardiogenesis in vertebrates	6.49E-04
	**Molecular and Cellular Functions**	**p-value**
	Cell death and survival	6.45E-03 – 2.10E-08
	Cellular movement	6.45E-03 – 1.24E-07
	Cellular growth and proliferation	6.48E-03 – 1.29E-06

## Data Availability

All the data supporting this study are available within the article and Supplementary information.

## References

[1] Siegel R. L., Miller K. D., Wagle N. S. & Jemal A. Cancer statistics, 2023. CA Cancer J Clin 73, 17–48, doi:10.3322/caac.21763 (2023). 36633525

[2] Damodaran S., Lang J. M. & Jarrard D. F. Targeting Metastatic Hormone Sensitive Prostate Cancer: Chemohormonal Therapy and New Combinatorial Approaches. J Urol 201, 876–885, doi:10.1097/JU.0000000000000117 (2019). 30747897

[3] Davis I. D. Enzalutamide with Standard First-Line Therapy in Metastatic Prostate Cancer. N Engl J Med 381, 121–131, doi:10.1056/NEJMoa1903835 (2019). 31157964

[4] Wala J., Nguyen P. & Pomerantz M. Early Treatment Intensification in Metastatic Hormone-Sensitive Prostate Cancer. J Clin Oncol 41, 3584–3590, doi:10.1200/JCO.23.00723 (2023). 37267579 PMC10325768

[5] Lundberg A. The genomic and epigenomic landscape of double-negative metastatic prostate cancer. Cancer Res, doi:10.1158/0008-5472.CAN-23-0593 (2023). PMC10425725 37289025

[6] Kneppers J. Extensive androgen receptor enhancer heterogeneity in primary prostate cancers underlies transcriptional diversity and metastatic potential. Nat Commun 13, 7367, doi:10.1038/s41467-022-35135-2 (2022). 36450752 PMC9712620

[7] Quigley D. A. Genomic Hallmarks and Structural Variation in Metastatic Prostate Cancer (vol 174, pg 758, 2018). Cell 175, 889–889, doi:10.1016/j.cell.2018.10.019 (2018). 30340047

[8] Dai C., Dehm S. M. & Sharifi N. Targeting the Androgen Signaling Axis in Prostate Cancer. J Clin Oncol, JCO2300433, doi:10.1200/JCO.23.00433 (2023). PMC10852396 37429011

[9] Baca S. C. Reprogramming of the FOXA1 cistrome in treatment-emergent neuroendocrine prostate cancer. Nat Commun 12, 1979, doi:10.1038/s41467-021-22139-7 (2021). 33785741 PMC8010057

[10] He Y. Targeting signaling pathways in prostate cancer: mechanisms and clinical trials. Signal Transduct Target Ther 7, 198, doi:10.1038/s41392-022-01042-7 (2022). 35750683 PMC9232569

[11] Schumacher F. R. Association analyses of more than 140,000 men identify 63 new prostate cancer susceptibility loci (vol 50, pg 928, 2018). Nat Genet 51, 363–363, doi:10.1038/s41588-018-0330-6 (2019). 29892016 PMC6568012

[12] Conti D. V. Trans-ancestry genome-wide association meta-analysis of prostate cancer identifies new susceptibility loci and informs genetic risk prediction. Nat Genet 53, 65–75, doi:10.1038/s41588-020-00748-0 (2021). 33398198 PMC8148035

[13] Yuan J. Prostate Cancer Transcriptomic Regulation by the Interplay of Germline Risk Alleles, Somatic Mutations, and 3D Genomic Architecture. Cancer Discov 12, 2838–2855, doi:10.1158/2159-8290.CD-22-0027 (2022). 36108240 PMC9722594

[14] Takata R. Genome-wide association study identifies five new susceptibility loci for prostate cancer in the Japanese population. Nat Genet 42, 751–754, doi:10.1038/ng.635 (2010). 20676098

[15] Lindstrom S. Replication of five prostate cancer loci identified in an Asian population--results from the NCI Breast and Prostate Cancer Cohort Consortium (BPC3). Cancer epidemiology, biomarkers & prevention : a publication of the American Association for Cancer Research, cosponsored by the American Society of Preventive Oncology 21, 212–216, doi:10.1158/1055-9965.EPI-11-0870-T (2012). PMC3253912 22056501

[16] Batra J. A replication study examining novel common single nucleotide polymorphisms identified through a prostate cancer genome-wide association study in a Japanese population. American journal of epidemiology 174, 1391–1395, doi:10.1093/aje/kwr271 (2011). 22085625

[17] Xu X. Variants at IRX4 as prostate cancer expression quantitative trait loci. European journal of human genetics : EJHG 22, 558–563, doi:10.1038/ejhg.2013.195 (2014). 24022300 PMC3953920

[18] Chen J. K. IRX5 promotes DNA damage repair and activation of hair follicle stem cells. Stem Cell Reports 18, 1227–1243, doi:10.1016/j.stemcr.2023.03.013 (2023). 37084727 PMC10202659

[19] Rodrigues M. Homeobox gene amplification and methylation in oral squamous cell carcinoma. Arch Oral Biol 129, 105195, doi:10.1016/j.archoralbio.2021.105195 (2021). 34126417

[20] Dos Santos E. C., Petrone I., Binato R. & Abdelhay E. Iroquois Family Genes in Gastric Carcinogenesis: A Comprehensive Review. Genes (Basel) 14, doi:10.3390/genes14030621 (2023). PMC10048635 36980893

[21] Nguyen H. H. IRX4 at 5p15 suppresses prostate cancer growth through the interaction with vitamin D receptor, conferring prostate cancer susceptibility. Human molecular genetics 21, 2076–2085, doi:10.1093/hmg/dds025 (2012). 22323358

[22] Chakma K. Epigenetic inactivation of IRX4 is responsible for acceleration of cell growth in human pancreatic cancer. Cancer Sci, doi:10.1111/cas.14644 (2020). PMC7734003 32894817

[23] Jia Z. 1,25-dihydroxyvitamin D3 signaling-induced decreases in IRX4 inhibits NANOG-mediated cancer stem-like properties and gefitinib resistance in NSCLC cells. Cell Death Dis 11, 670, doi:10.1038/s41419-020-02908-w (2020). 32820157 PMC7441324

[24] Correa S. Identifying potential markers in Breast Cancer subtypes using plasma label-free proteomics. Journal of proteomics 151, 33–42, doi:10.1016/j.jprot.2016.07.030 (2017). 27498391

[25] Shah N. & Sukumar S. The Hox genes and their roles in oncogenesis. Nature reviews. Cancer 10, 361–371, doi:10.1038/nrc2826 (2010). 20357775

[26] Bhatlekar S., Fields J. Z. & Boman B. M. HOX genes and their role in the development of human cancers. Journal of molecular medicine 92, 811–823, doi:10.1007/s00109-014-1181-y (2014). 24996520

[27] Rhodes D. R. ONCOMINE: a cancer microarray database and integrated data-mining platform. Neoplasia 6, 1–6 (2004). 15068665 10.1016/s1476-5586(04)80047-2 PMC1635162

[28] Liu P. Sex-determining region Y box 4 is a transforming oncogene in human prostate cancer cells. Cancer Res 66, 4011–4019, doi:10.1158/0008-5472.CAN-05-3055 (2006). 16618720

[29] Wallace T. A. Tumor immunobiological differences in prostate cancer between African-American and European-American men. Cancer Res 68, 927–936, doi:10.1158/0008-5472.CAN-07-2608 (2008). 18245496

[30] Taylor B. S. Integrative genomic profiling of human prostate cancer. Cancer Cell 18, 11–22, doi:10.1016/j.ccr.2010.05.026 (2010). 20579941 PMC3198787

[31] Grasso C. S. The mutational landscape of lethal castration-resistant prostate cancer. Nature 487, 239–243, doi:10.1038/nature11125 (2012). 22722839 PMC3396711

[32] Arredouani M. S. Identification of the transcription factor single-minded homologue 2 as a potential biomarker and immunotherapy target in prostate cancer. Clinical cancer research : an official journal of the American Association for Cancer Research 15, 5794–5802, doi:10.1158/1078-0432.CCR-09-0911 (2009). 19737960 PMC5573151

[33] Vanaja D. K., Cheville J. C., Iturria S. J. & Young C. Y. Transcriptional silencing of zinc finger protein 185 identified by expression profiling is associated with prostate cancer progression. Cancer Res 63, 3877–3882 (2003). 12873976

[34] Duverger O. & Morasso M. I. Role of homeobox genes in the patterning, specification, and differentiation of ectodermal appendages in mammals. Journal of cellular physiology 216, 337–346, doi:10.1002/jcp.21491 (2008). 18459147 PMC2561923

[35] Paltoglou S. Novel Androgen Receptor Coregulator GRHL2 Exerts Both Oncogenic and Antimetastatic Functions in Prostate Cancer. Cancer Res 77, 3417–3430, doi:10.1158/0008-5472.CAN-16-1616 (2017). 28473532 PMC5497757

[36] Stelloo S. Endogenous androgen receptor proteomic profiling reveals genomic subcomplex involved in prostate tumorigenesis. Oncogene, doi:10.1038/onc.2017.330 (2017). 28925401

[37] Yu J. An integrated network of androgen receptor, polycomb, and TMPRSS2-ERG gene fusions in prostate cancer progression. Cancer Cell 17, 443–454, doi:10.1016/j.ccr.2010.03.018 (2010). 20478527 PMC2874722

[38] Bu H. Putative Prostate Cancer Risk SNP in an Androgen Receptor-Binding Site of the Melanophilin Gene Illustrates Enrichment of Risk SNPs in Androgen Receptor Target Sites. Human mutation 37, 52–64, doi:10.1002/humu.22909 (2016). 26411452 PMC4715509

[39] Dadaev T. Fine-mapping of prostate cancer susceptibility loci in a large meta-analysis identifies candidate causal variants. Nat Commun 9, 2256, doi:10.1038/s41467-018-04109-8 (2018). 29892050 PMC5995836

[40] Penney K. L. Association of Prostate Cancer Risk Variants with TMPRSS2:ERG Status: Evidence for Distinct Molecular Subtypes. Cancer epidemiology, biomarkers & prevention : a publication of the American Association for Cancer Research, cosponsored by the American Society of Preventive Oncology 25, 745–749, doi:10.1158/1055-9965.EPI-15-1078 (2016). PMC4873420 26941365

[41] Cai C. ERG induces androgen receptor-mediated regulation of SOX9 in prostate cancer. The Journal of clinical investigation 123, 1109–1122, doi:10.1172/JCI66666 (2013). 23426182 PMC3582143

[42] Fernando A., Liyanage C., Moradi A., Janaththani P. & Batra J. Identification and Characterization of Alternatively Spliced Transcript Isoforms of IRX4 in Prostate Cancer. Genes (Basel) 12, doi:10.3390/genes12050615 (2021). PMC8143155 33919200

[43] Liyanage C., Fernando A. & Batra J. Differential roles of protease isoforms in the tumor microenvironment. Cancer metastasis reviews 38, 389–415, doi:10.1007/s10555-019-09816-2 (2019). 31673830

[44] Chandra S., Srinivasan S. & Batra J. Hepatocyte nuclear factor 1 beta: A perspective in cancer. Cancer Med 10, 1791–1804, doi:10.1002/cam4.3676 (2021). 33580750 PMC7940219

[45] Nelson D. O. Irx4 Marks a Multipotent, Ventricular-Specific Progenitor Cell. Stem cells 34, 2875–2888, doi:10.1002/stem.2486 (2016). 27570947 PMC5123941

[46] Myrthue A. The iroquois homeobox gene 5 is regulated by 1,25-dihydroxyvitamin D3 in human prostate cancer and regulates apoptosis and the cell cycle in LNCaP prostate cancer cells. Clinical cancer research : an official journal of the American Association for Cancer Research 14, 3562–3570, doi:10.1158/1078-0432.CCR-07-4649 (2008). 18519790

[47] Lee Y. Y. CSF2 Overexpression Is Associated with STAT5 Phosphorylation and Poor Prognosis in Patients with Urothelial Carcinoma. Journal of Cancer 7, 711–721, doi:10.7150/jca.14281 (2016). 27076853 PMC4829558

[48] Yang Y. A. & Yu J. Current perspectives on FOXA1 regulation of androgen receptor signaling and prostate cancer. Genes Dis 2, 144–151, doi:10.1016/j.gendis.2015.01.003 (2015). 26114156 PMC4477823

[49] Pomerantz M. M. The androgen receptor cistrome is extensively reprogrammed in human prostate tumorigenesis. Nat Genet 47, 1346–1351, doi:10.1038/ng.3419 (2015). 26457646 PMC4707683

[50] Tan M. H., Li J., Xu H. E., Melcher K. & Yong E. L. Androgen receptor: structure, role in prostate cancer and drug discovery. Acta pharmacologica Sinica 36, 3–23, doi:10.1038/aps.2014.18 (2015). 24909511 PMC4571323

[51] Whitington T. Gene regulatory mechanisms underpinning prostate cancer susceptibility. Nat Genet 48, 387–397, doi:10.1038/ng.3523 (2016). 26950096

[52] Tsunoda T. & Takagi T. Estimating transcription factor bindability on DNA. Bioinformatics 15, 622–630 (1999). 10487870 10.1093/bioinformatics/15.7.622

[53] Bruneau B. G. Cardiac expression of the ventricle-specific homeobox gene Irx4 is modulated by Nkx2-5 and dHand. Developmental biology 217, 266–277, doi:10.1006/dbio.1999.9548 (2000). 10625552

[54] Cerami E. The cBio cancer genomics portal: an open platform for exploring multidimensional cancer genomics data. Cancer discovery 2, 401–404, doi:10.1158/2159-8290.CD-12-0095 (2012). 22588877 PMC3956037

[55] Nelson P. S. The program of androgen-responsive genes in neoplastic prostate epithelium. Proc Natl Acad Sci U S A 99, 11890–11895, doi:10.1073/pnas.182376299 (2002). 12185249 PMC129364

